# Multimodal neuroimaging data from a 5-week heart rate variability biofeedback randomized clinical trial

**DOI:** 10.1038/s41597-023-02396-5

**Published:** 2023-07-29

**Authors:** Hyun Joo Yoo, Kaoru Nashiro, Jungwon Min, Christine Cho, Noah Mercer, Shelby L. Bachman, Padideh Nasseri, Shubir Dutt, Shai Porat, Paul Choi, Yong Zhang, Vardui Grigoryan, Tiantian Feng, Julian F. Thayer, Paul Lehrer, Catie Chang, Jeffrey A. Stanley, Elizabeth Head, Jeremy Rouanet, Vasilis Z. Marmarelis, Shrikanth Narayanan, Jessica Wisnowski, Daniel A. Nation, Mara Mather

**Affiliations:** 1 University of Southern California, Los Angeles, CA 90007 USA; 2 University of California, Irvine, USA; 3 Rutgers University, New Brunswick–Piscataway, USA; 4 Vanderbilt University, Nashville, USA; 5 Wayne State University School of Medicine, Detroit, USA

**Keywords:** Emotion, Human behaviour, Cognitive ageing

## Abstract

We present data from the Heart Rate Variability and Emotion Regulation (HRV-ER) randomized clinical trial testing effects of HRV biofeedback. Younger (N = 121) and older (N = 72) participants completed baseline magnetic resonance imaging (MRI) including T_1_-weighted, resting and emotion regulation task functional MRI (fMRI), pulsed continuous arterial spin labeling (PCASL), and proton magnetic resonance spectroscopy (^1^H MRS). During fMRI scans, physiological measures (blood pressure, pulse, respiration, and end-tidal CO_2_) were continuously acquired. Participants were randomized to either increase heart rate oscillations or decrease heart rate oscillations during daily sessions. After 5 weeks of HRV biofeedback, they repeated the baseline measurements in addition to new measures (ultimatum game fMRI, training mimicking during blood oxygen level dependent (BOLD) and PCASL fMRI). Participants also wore a wristband sensor to estimate sleep time. Psychological assessment comprised three cognitive tests and ten questionnaires related to emotional well-being. A subset (N = 104) provided plasma samples pre- and post-intervention that were assayed for amyloid and tau. Data is publicly available via the OpenNeuro data sharing platform.

## Background & Summary

Heart rate variability (HRV) is one of the most consistent correlates of psychological and emotional well-being and stress^1–3^. However, it is not just random variation in the interval between heartbeats that is associated with well-being. In healthy resting people, heart rate is tonically suppressed by signals transmitted via the vagus nerve. This suppression of heart rate is stronger when exhaling than when inhaling^4^, and it is “vagal HRV” or the high frequency oscillations in heart rate in response to breathing that are most strongly associated with positive well-being (or with low negative affect or stress). Spending time every day breathing at a pace of around 10 seconds per breath (a pace that induces resonance with the baroreflex and so induces high oscillations in heart rate) while getting biofeedback on heart rate oscillatory activity can enhance well-being^5,6^. This suggests that heart rate oscillatory activity serves as more than a readout of the integrity of the brain’s autonomic regulatory systems. Short bouts of high heart rate oscillations may stimulate these regulatory systems, enhancing their function^7^. To test this hypothesis, in a randomized clinical trial (ClinicalTrials.gov NCT03458910), we scanned younger and older participants while at rest and while doing an emotion regulation task both before and after five weeks of daily practice sessions in which they received heart rate variability biofeedback to either increase (Osc+ condition) or decrease (Osc- condition) heart rate oscillations.

Initial studies using heart rate variability biofeedback yielded promising results for self-reported emotion and there has been a significant growth in research on this intervention^5,6^. Compared with most prior HRV-biofeedback studies, our study has a larger number of participants and a more extensive set of outcome measures. It is also unique among HRV-biofeedback studies in having all the following features: functional and structural brain outcome measures, a well-matched active comparison group, inclusion of two age groups, and heart rate data available from each practice session. Thus, these Heart Rate Variability and Emotion Regulation (HRV-ER) clinical trial data should be a rich source for a variety of secondary analyses, including those investigating individual-difference factors that affect responses to HRV-biofeedback, examination of age differences in response to the intervention, and specific patterns of brain changes in response to the intervention. Furthermore, the baseline pre-intervention data could be relevant for potential secondary analyses unrelated to heart rate biofeedback. For instance, the larger N than seen in most fMRI emotion regulation studies allows for individual difference comparisons, especially given all the additional physiological data collected from each participant. In addition, this study includes data not typically available in public datasets, such as PCASL, a turbo spin echo (TSE) structural sequence targeting the locus coeruleus, biochemical measurements using proton magnetic resonance spectroscopy (¹H MRS), and plasma amyloid and tau levels in both younger and older adults, allowing for unique secondary analyses not previously feasible.

This dataset has already been a rich source of interesting findings, as reported in our recent publications^8–15^. The younger adults’ baseline emotion regulation task data revealed that the implicit assumption in the field that amplifying vs. diminishing emotions acts on the same affect-related brain regions was incorrect^10^. Upregulation increased activity in brain regions associated with emotional experience, such as the amygdala, anterior insula, striatum, and anterior cingulate gyrus. In contrast, downregulation decreased activity in regions receiving interoceptive input, such as the posterior insula and postcentral gyrus. In subsequent analyses of both pre- and post-intervention data, we found that Osc+ participants decreased activity in these interoception-related regions more when trying to diminish their emotional response to pictures than they had before the intervention, whereas the Osc- participants did not show these changes^12^. The Osc+ intervention also increased functional connectivity between the left amygdala and mPFC and within canonical emotion-related brain networks, whereas the Osc- condition did not affect these functional connectivity measures^12^. Emotional memory was more positively biased in the Osc+ than Osc- condition, an effect mediated by change in left amygdala-mPFC functional connectivity^9^. Furthermore, the two interventions affected structural volume in opposing directions in the left orbitofrontal cortex^15^, the region in which we previously had found individual differences in structure to be associated with resting vagal HRV^16,17^. Partway through the clinical trial (see ‘Study Timeline and Implications’), we added blood draws to the pre- and post-intervention assessment protocols to allow for exploratory investigation of how manipulating heart rate oscillations affects plasma amyloid and tau levels, a question motivated by links between Alzheimer’s disease pathways and factors associated with autonomic system function, such as stress^18^ and noradrenergic activity^19^ We found that, in both younger and older adults, the Osc+ intervention reduced plasma amyloid β levels whereas the Osc- intervention increased plasma amyloid β levels^11^. In summary, our findings indicate that daily biofeedback to increase or decrease heart rate oscillations affects brain activity and structure in brain regions associated with emotion and HRV, while also affecting levels of amyloid β in the periphery. It is likely that HRV biofeedback stimulates multiple interacting pathways that lead to these and other effects. We hope that making this dataset freely accessible to researchers will accelerate understanding of autonomic factors influencing the brain and body and development of interventions that use such knowledge to improve health and well-being.

## Methods

### Power considerations for sample size

When planning this study, no prior studies had examined effects of these interventions on brain function so we were unable to estimate effect sizes based on prior neuroimaging data. We elected to power our study to detect medium or larger effect sizes. Our main planned statistical comparisons were repeated-measures ANOVAs with within-between interactions. For these, a total sample size of 46 would give 90% power to detect moderate effect sizes of f = 0.25 with *α* = 0.05, given an assumed correlation among the repeated measures of 0.5^20^. We also planned to examine within-subject change within each of the conditions. A sample size of 44 in each group would give 90% power to detect within-group change effect sizes of d = 0.5 in a two-tailed t-test with *α* = 0.05^20^. Thus, we aimed for an N = 100 completion rate across the two intervention conditions for each age group to be able to accommodate potential exclusions for movement during imaging or other data quality issues.

### Participants

**Fig. 1:**
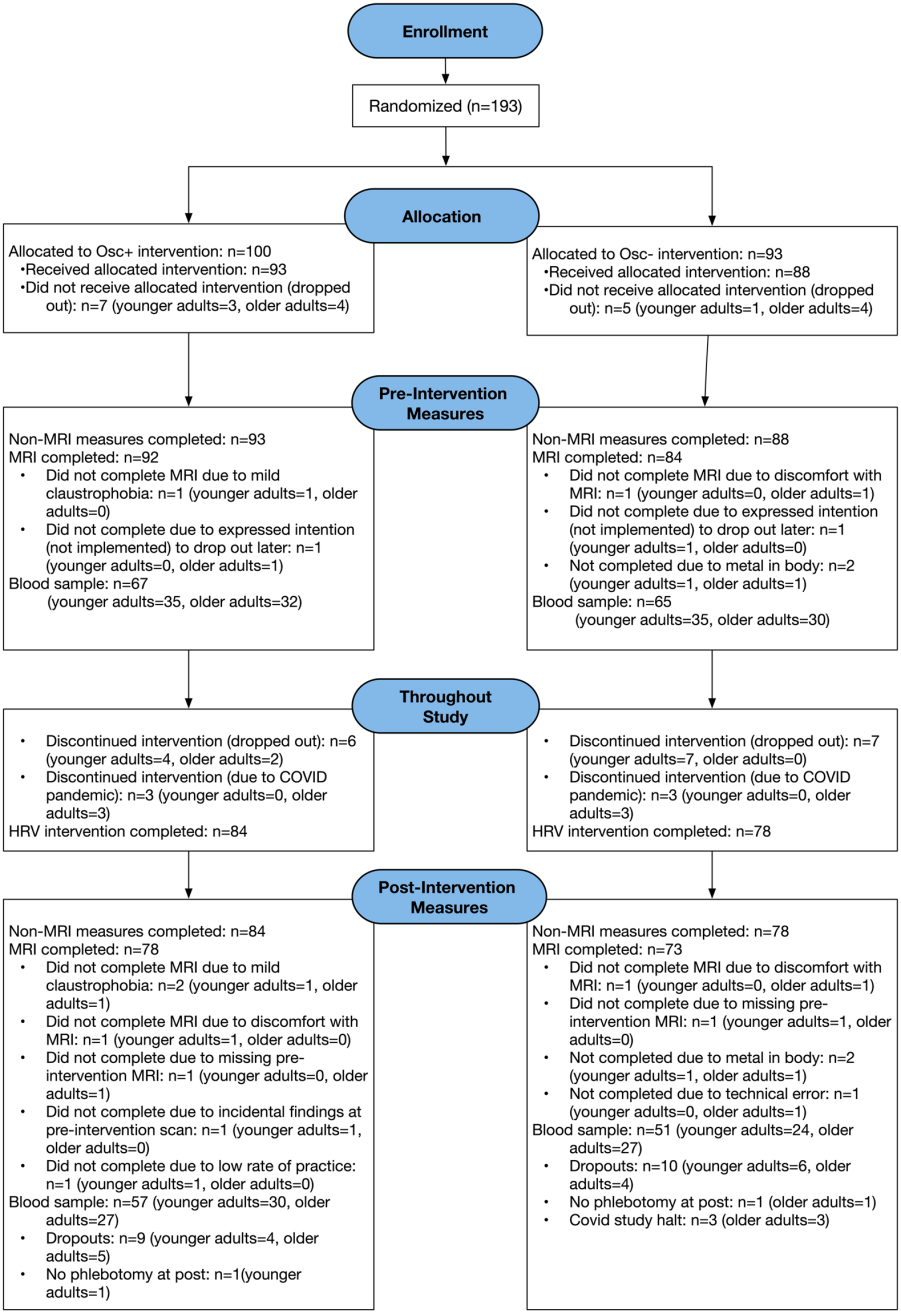
CONSORT flow diagram showing participant flow through each stage of the randomized controlled trial.

We recruited 121 younger participants aged between 18 and 35 years and 72 older participants aged between 55 and 80 years via the USC Healthy Minds community subject pool, a USC online bulletin board, Facebook and flyers (see Fig. 1 for drop-out rates per condition). Participants provided informed consent approved by the University of Southern California (USC) Institutional Review Board. Participants were recruited in waves of approximately 20 participants from the same age group, each of whom was assigned to a small group of 3–6 people. Groups met for weekly lab visits at the same time and day each week. After group assignments of a wave were complete, we assigned each group to one of two conditions involving daily biofeedback that aimed to increase heart rate oscillations (Osc+ condition) or decrease heart rate oscillations (Osc- condition). To maintain balanced numbers in each condition we determined how many groups had been assigned to a condition in the previous wave; for example, if 2 out of 5 groups in a previous wave were assigned the Osc+ condition, then 3 out of 5 groups would be assigned that condition in the next wave. One research staff member who was blinded to participants and small group assignment generated the random numbers and assigned the conditions to each small group. The study utilized a single-blinded design; the consent document did not mention that there were two conditions and participants in both conditions were told that we were interested in how training to control heart rate might influence emotional health and the functions of brain regions involved in emotion regulation. Upon completing the study, participants were paid for their participation and received bonus payments based on their individual and group performances. Participants had a chance to earn individual performance rewards based on their weekly performance. They could earn $2 for each instance they exceeded their assigned target score, with a maximum limit of 10 instances. Additionally, group performance rewards were available if the members of a participant’s group completed at least 80% of their assigned biofeedback training minutes. The rewards were calculated on a weekly basis, and participants received updates on their earnings during their lab visit. Prospective participants were screened and excluded for major medical, neurological, or psychiatric illnesses. We excluded people who had a disorder that would impede performing the HRV biofeedback procedures (e.g., coronary artery disease, angina, cardiac pacemaker), who currently were training using a relaxation, biofeedback or breathing practice, or were on any psychoactive drugs other than antidepressants or anti-anxiety medications. We included people who were taking antidepressant or anti-anxiety medication and/or attending psychotherapy only if the treatment had been ongoing and unchanged for at least three months and no changes were anticipated. Gender, education, age, and race did not differ significantly in the two conditions.

### Study timeline and implications

Recruitment and enrollment occurred between February 2018 and March 2020. We started recruitment for the younger cohort first, as we aimed to have completed data from the younger adults to analyze and report on while we recruited and ran the older adult cohort. Based on our experience with the younger cohort and initial testing with older adults, we made a few minor changes to the protocol for older adults (we cut out one stress task and slightly modified the Ultimatum Game task; see Methods for more information). Partway through data collection for younger adults, we added blood draws to the pre- and post-intervention visits. Thus, about half the younger and all the older adults were asked to provide blood samples. Older adult enrollment was cut short by the COVID pandemic.

### Overview of 7-week protocol schedule

**Fig. 2:**
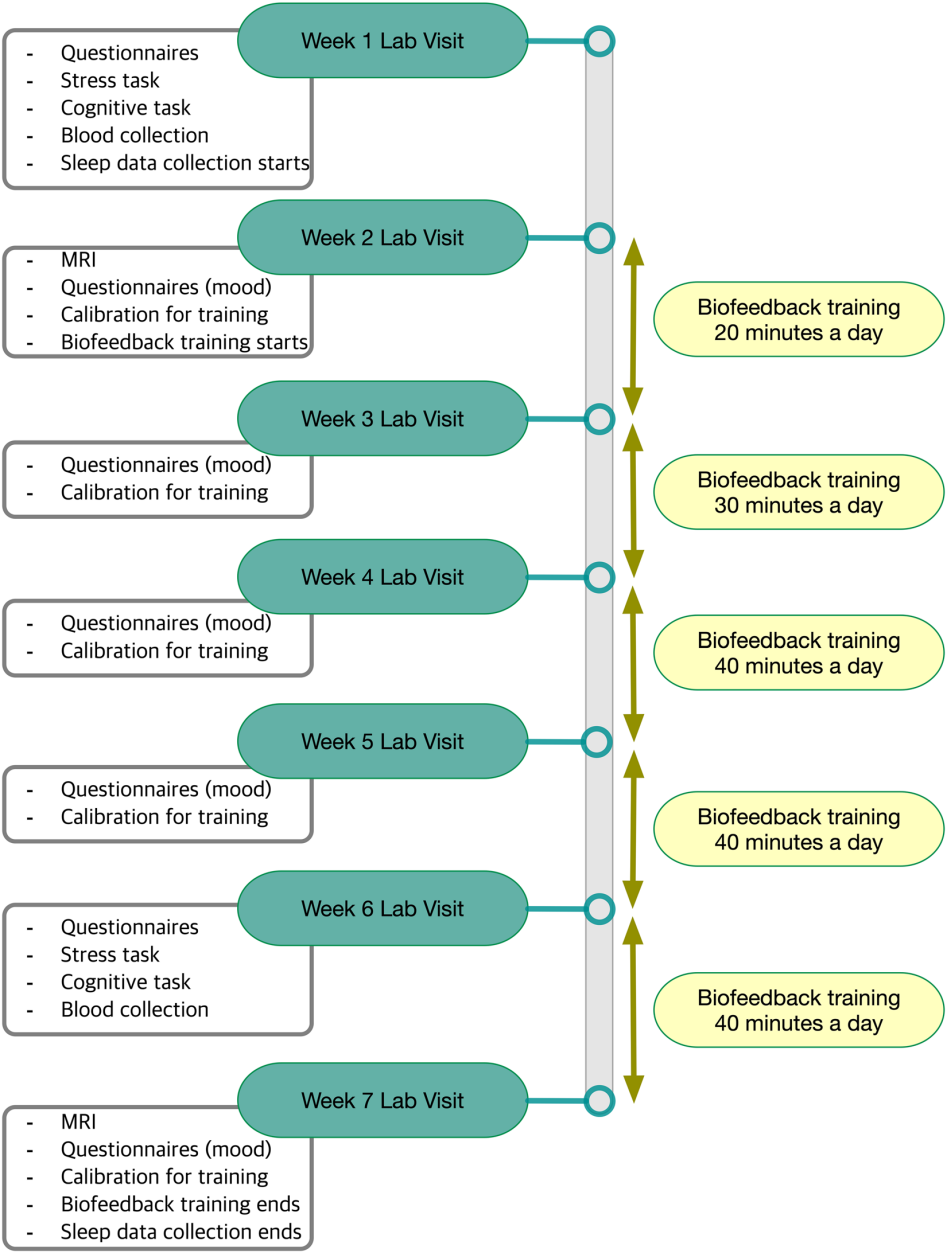
Study 7-week schedule.

As previously detailed^12^, the study protocol involved seven weekly lab visits and five weeks of home biofeedback training (Fig. 2). Each lab visit began with questionnaires assessing mood and anxiety. The first lab visit involved non-MRI baseline measurements, including several questionnaires. The second lab visit involved the baseline MRI session, followed by the first biofeedback calibration and training session (see below for details). The weekly lab visits (except for weeks with MRI sessions) were run in small groups in which participants shared their experiences and tips about biofeedback training with other participants from the same condition, while 1–2 researchers facilitated the discussion. Outside the lab, participants used a customized social app to communicate with other group members and researchers about their progress on daily biofeedback training. The Week-6 lab visit repeated the assessments from the first lab visit. The final (7th) lab visit repeated the baseline MRI session scans in the same order. Additional training-session scans were collected at the end of the scan protocol. Finally, after the scan, participants completed a post-study questionnaire. Table 1 provides detailed information about the measurement at each time point.

**Table 1:** Measurement at each time point.

Category	Data type	Week1	Week2	Week3	Week4	Week5	Week6	Week7	Measurement
Demographics	Demographics	*							•Self-report
Questionnaires	Emotion (POMS, SAI)	*	*	*	*	*	*	*	•Self-report
Questionnaires	Emotion (CESD, TAI)	*	*				*	*	•Self-report
Questionnaires	Altruism		*						•Self-report
Questionnaires	Others (e.g., FFMQ)	*					*		•Self-report
Cognitive task	NIH toolbox: Cognition	*					*		•Score
Cognitive task	SART	*					*		•Score •response time
Cognitive task	Picture memory task				*	*			•Encoding (Week-4) •Recognition (Week-5) •Recall (Week-5)
Heart rate data	Calibration		*	*	*	*	*	*	•Inter-beat-interval data from pulse
Heart rate data	Home training		*	*	*	*	*		•Inter-beat-interval data from pulse
Stress task	Physiological data	*					*		•ECG, respiration, GSR, continuous blood pressure
Stress task	Behavioral data	*					*		•Score
Blood sample	Blood plasma sample	*					*		•Aβ42, Aβ40, tTau, and pTau-181
MR Imaging	Functional-resting		*					*	•Brain imaging data •Physiological data
MR Imaging	Functional-resting ASL		*					*	•Brain imaging data •Physiological data
MR Imaging	Functional-Emotion regulation		*					*	•Brain imaging data •Physiological data •Event data
MR Imaging	Anatomical-T_1_		*					*	•Brain imaging data
MR Imaging	^1^H MRS		*					*	•Brain biochemistry data
MR Imaging	Anatomical-TSE		*					*	•Brain imaging data (locus coeruleus)
MR Imaging	Functional-UG							*	•Brain imaging data •Physiological data •Event data
MR Imaging	Functional-training mimicking							*	•Brain imaging data •Physiological data •Event data
MR Imaging	Functional-training mimicking ASL							*	•Brain imaging data •Physiological data •Event data

### Biofeedback training

#### Osc+ Group

Participants wore an infrared pulse plethysmograph^21^ ear sensor to measure their pulse. They viewed real-time heart rate biofeedback while breathing in through the nose and out through the mouth in synchrony with a visual pacer. The software^22^ provided a summary ‘coherence’ score for participants calculated as peak power/(total power – peak power). Peak power was determined by finding the highest peak within the range of 0.04–0.26 Hz and calculating the integral of the window 0.015 Hz above and below this highest peak. Total power was computed for the 0.0033–0.4 Hz range^12^.

During the second lab visit, we introduced participants to the device and had them complete five minutes of rest followed by 5-min paced breathing segments at 9 s, 10 s, 11 s, 12 s, and 13 s per breath, which approximately correspond with the 6.5, 6, 5.5, 5 and 4.5 breaths/min paces in Lehrer *et al*.^23^. Next, we computed various aspects of the oscillatory dynamics for each breathing pace using Kubios HRV Premium 3.1 software^24^ and assessed which breathing pace appeared to best approximate their resonance frequency^23^ and to maximize their coherence scores. For more detail, see Nashiro *et al*.^12^. Participants were then instructed to train at home with the pacer set to this frequency.

During the subsequent weekly visits, the participants completed a 5-min rest segment followed by paced breathing segments to calibrate the best resonance frequency^12^.

#### Osc- Group

This condition was designed to be as similar as possible to the Osc+ condition, but with the opposing goal (to reduce heart rate oscillations). The same biofeedback ear sensor device was used in this condition and participants were asked to practice for the same amount of time. However, we created custom software to display a different set of feedback to the Osc- participants^25^. During each Osc- training session, a ‘calmness’ score was provided as feedback to the participants instead of the coherence score (see Nashiro *et al*. (2023) for details)^12^.

### Heart rate data from lab calibration and home training

During lab calibration, pulse was measured using HeartMath emWave pro software with an infrared pulse plethysmograph (PPG) ear sensor while participants sat in a chair with knees at a 90 degrees angle and both feet flat on the floor. The pulse wave was recorded with a sampling rate of 370 Hz. Interbeat interval (IBI) data was extracted after eliminating ectopic beats and other artifactual signals through a built-in process in emWave pro. On each home training session, pulse was measured using the same devices and software used for calibration sessions. An IBI data file was saved on a study-provided laptop and transferred to the lab server after completing each session.

### Questionnaires

#### Emotion questionnaires

During each lab visit, participants completed the profile of mood states (POMS)^26^ and the State Anxiety Inventory (SAI)^27^. We used the 40-item version of POMS. Participants reported how much each item reflected how they felt at the moment using a scale from 1 (not at all) to 5 (extremely). Total mood disturbance was calculated by subtracting positive-item totals from negative-item totals. A constant value (i.e. 100) was added to the total mood disturbance to eliminate negative scores. The SAI measures state anxiety using 20 statements. Participants indicated how they felt at the moment on a scale from 1 (not at all) to 4 (very much so). In Weeks 1, 2, 6 and 7, we also administered the Trait Anxiety Inventory (TAI)^27^ and the Center for Epidemiological Studies Depression Scale (CES-D) in Weeks 1, 2, 6 and 7^28^. The TAI measures trait anxiety using 20 statements, which participants rated on a 4-point scale from 1 (not at all) to 4 (very much so). The CES-D consists of 20 statements, which participants rated on a 4-point scale from 0 (rarely) to 3 (most or all of the time). At Week-1 and Week-6 lab visits, participants completed six additional emotion questionnaires. We previously reported SAI, POMS, and CESD data for younger participants^12^ and younger and older participants^11^.

We assessed trait mindfulness using the 20-item version of the Five Facet Mindfulness Questionnaire (FFMQ)^29^. Participants rated each item using a scale from 1 (never) to 5 (very often/always true). We also administered the Smith Relaxation States Inventory 3 (SRSI3)^30^ to assess various aspects of stress, relaxation, meditation, and mindfulness. Participants completed state and disposition versions of the SRSI3 each consisting of 38 items. The state version asks how you “feel right now” on a 6-point scale from 1 (not at all) to 6 (maximum). The disposition version asks how often you experience relaxation states and stress states. We slightly modified the disposition version and asked how often each item has been experienced “in the past month” on a 6-point scale from 1 (rarely or never, less than once a month) to 6 (a lot, more than once a day). We calculated SRSI3 scores based on the Relaxation/Meditation/Mindfulness (RMM) Tracker/SRSI3 Manual v9.9.2020, which includes 34 items for scoring^31^. We measured the extent and severity of fatigue using the 11-item Chalder Fatigue Scale (CFQ 11)^32^ on a 4-point scale from 0 (less than usual) to 3 (much more than usual) or 0 (better than usual) to 3 (much worse than usual). We also administered the 10-item Emotion Regulation Questionnaire (ERQ)^33^, which is designed to measure the tendency to regulate emotions in two ways (cognitive reappraisal and expressive suppression) on a 7-point scale from 1 (strongly disagree) to 7 (strongly agree). Participants also completed a self-efficacy version of the 10-item ERQ, which asks how “capable” they are of regulating their emotions on the same 7-point scale. In addition, perceived stress was measured using the NIH Toolbox Perceived Stress Survey^34^, a 10-item version of the Perceived Stress Scale^35^. Participants rated the frequency of stressful experiences and the extent to which they felt strained or overloaded during the past month (e.g., How often have you felt nervous and “stressed”? How often have you felt difficulties were piling up so high that you could not overcome them?) on a five-point scale, ranging from Never (1) to Very Often (5); higher scores correspond to greater perceived stress. We calculated the score on the perceived stress scale using the mean score based on the NIH toolbox scoring method^36^.

We calculated descriptive statistics and reliability estimates of each subscale of the emotion questionnaire (Table 2). We reported average Cronbach’s alpha coefficients from multiple time-points to provide information about their internal consistency.

**Table 2:** Summary of internal consistency (Cronbach’s alpha) and test-retest reliability estimates in emotion questionnaires.

Abbr.	Full Name	Reference	No. of Items	Construct	M (SD)	Cronbach’s alpha	Intraclass correlation coefficient	Typical percentage error (%)^35^
POMS	The profile of mood states	Grove & Prapavessis (1992)^25^	40	Total Mood Disturbance	88.46 (14.4)	0.88 (0.85–0.91)	0.67	11.02
SAI	The state anxiety inventory	Spielberger *et al*.^26^	20	State anxiety	36.33 (9.36)	0.72 (0.59–0.77)	0.70	15.92
TAI	The trait anxiety inventory	Spielberger *et al*.^26^	20	Trait anxiety	38.46 (10.04)	0.55 (0.46–0.61)	0.71	14.86
CESD	The Center for Epidemiological Studies Depression Scale	Radloff (1977)^27^	20	Depression	12.44 (7.74)	0.71 (0.67–0.74)	0.71	37.33
FFMQ	The Five Facet Mindfulness Questionnaire	Tran *et al*.^28^	20	Mindfulness	67.98 (10.33)	0.65 (0.60–0.70)	0.82	6.64
SRSI3-I	The Smith Relaxation States Inventory 3-I	Smith (2001)^29^	38 (26) 38 (8)	Relaxation: state Stress: state	3.11 (0.84) 1.81 (0.64)	0.94 (0.93–0.95) 0.83 (0.82–0.83)	0.71 0.56	15.41 26.16
SRSI3-II	The Smith Relaxation States Inventory 3-II	Smith (2001)^29^	38 (26) 38 (8)	Relaxatio: frequency Stress: frequency	3.78 (0.85) 3.1 (0.96)	0.93 (0.927–0.932) 0.82 (0.818–0.826)	0.66 0.72	13.83 17.33
CFQ 11	The Chalder Fatigue Scale	Jackson (2015)^31^	11	Fatigue	12.48 (4.46)	0.86 (0.85–0.87)	0.56	25.53
ERQ-FR	The Emotion Regulation Questionnaire: frequency	Gross & John (2003)^32^	10	Emotion regulation: frequency	45.40 (7.24)	0.75 (0.74–0.76)	0.67	10.15
ERQ-SE	The Emotion Regulation Questionnaire: self-efficacy	Gross & John (2003)^32^	10	Emotion regulation: self-efficacy	49.92 (8.96)	0.88 (0.86–0.90)	0.55	13.52
PSS-10	Perceived stress scale	Cohen *et al*.^34^	10	Perceived stress	2.65 (0.59)	0.86 (0.84–0.89)	0.61	14.61

#### Altruism questionnaire

Participants completed the Altruism Scale Questionnaire^37^ during their Week-3 lab visits. The Altruism Scale Questionnaire is a self-report scale with 20 items each describing an altruistic behavior (e.g., “I have done volunteer work for a charity’’ and “I have delayed an elevator and held the door open for a stranger). Participants were instructed to rate the frequency of engaging in these behaviors on a 5-point scale (1 = never; 2 = once, 3 = more than once; 4 = often; 5 = very often). Higher scores in this scale correspond with higher altruistic tendencies.

#### Demographics and post-study questionnaire

During the Week-1 visit, participants completed questionnaires including basic demographics, clinical history including medications.

After the Week-7 post-intervention scan, participants completed a questionnaire surveying their experience during the study. They provided self-ratings of difficulty of daily heart rate biofeedback training, level of effort to complete the training, expectations of the training impact on well-being, and likelihood of continuing the training after the study’s conclusion.

### Stress task

The details of the stress task were reported previously^8^. During the Week-1 and Week-6 lab visits, participants completed a task designed to assess reactivity to and recovery from acute stress. The task consisted of several phases: a 4-minute baseline resting phase, a stress phase, and a 4-minute recovery resting phase. Younger participants completed two computerized tasks during the stress phase: a Paced Auditory Serial Addition Task (PASAT) and a modified Stroop color-word matching task. For more detail, see Bachman *et al*.^8^.

Physiological signals were recorded during all phases of the stress task using a BIOPAC MP160 system at a sampling frequency of 2 KHz. Electrocardiogram (ECG) and respiration signals were sent to the MP160 with a BioNomadix wireless transmitter. Blood pressure signals were measured from the non-dominant arm with a BIOPAC noninvasive blood pressure monitoring system (NIBP100D). For electrodermal activity recordings, disposable, pre-gelled Ag/AgCl electrodes (EL507) were attached to the palmar side of the medial phalange of the fourth and fifth fingers of each participant’s non-dominant hand (as the index and middle fingers were used for continuous blood pressure recordings). Raw physiological signals were split into segments corresponding to the phases of the stress task. For more detail, see Bachman *et al*.^8^.

### Cognitive tasks

#### The National Institutes of Health (NIH) toolbox cognitive battery

The NIH Toolbox Cognitive Battery is a component of the NIH Toolbox for Assessment of Neurological and Behavioral Function (www.nihtoolbox.org^38,39^) that comprises extensively validated computer-administered cognitive tests for use across childhood and adolescence, early adulthood, and old age. As detailed previously^14^, we administered the NIH-Toolbox cognitive battery using an iPad app on a 9.7 inch iPad Air 2. The Flanker Test, the List Sorting Working Memory (LSWM) Test, and the Pattern Comparison Processing Speed (PCPS) Test were administered to evaluate attention and executive function, working memory, and processing speed, respectively. For more detail, see Nashiro *et al*.^14^.

#### Sustained attention to response task (SART)

The SART^40^ was administered during Week-1 and Week-6 lab visits. During the task, participants were presented with a random series of single-digit numbers, ranging from 1 to 9. Participants were instructed to press the spacebar as soon as they saw each number other than 3. The task consisted of 225 trials where a single digit was presented for 250 ms with a 900-ms-lasting mask image between trials. The task took about 6 min and was based on the web-based Inquisit SART task developed by Millisecond Software^41^.

#### Picture memory tasks

As previously reported^9^, the emotional memory task was administered at the Week-4 and Week-5 lab visits. Seventy-two stimuli were selected from The Nencki Affective Picture System (NAPS)^42^, a database of realistic photographs that aim to induce positive, negative, or neutral emotional states. Stimuli were first counterbalanced by valence (24 each of positive, negative, and neutral); then two sets of 36 stimuli were created and counterbalanced by valence in each set (12 each of positive, negative, and neutral). Participants completed the task on the Qualtrics Survey platform. At the Week-4 visit, the encoding and immediate free recall task were administered and at the Week-5 visit, participants completed the same free recall task followed by a recognition task. For more detail, see Cho *et al*.^9^.

### Sleep time

Sleep and HRV derived from slow-wave sleep were measured with WHOOP wristbands^43^. Participants were provided WHOOP wristbands on the first and asked to wear them as close to 24 hours per day as possible until the final week of the study. All participants were instructed to wear the wristband as close to 24 hours per day as possible. For more detail, see Min *et al*.^11^. WHOOP algorithms have been validated by independent researchers as having a 95% sensitivity for sleep, 68% sensitivity for deep sleep and 70% for REM sleep^44^.

### MRI/MRS Data acquisition

The methods of resting-state during BOLD fMRI and pCASL, and emotion regulation task during fMRI were reported previously^12^, as were the methods of the T_1_-weighted structural scans^15^, and the methods of the T_1_-weighted TSE scan^8^. T1-weighted scans were defaced to ensure proper anonymization using PyDeface 2.0.0 after DICOM to NIfTI conversion.

#### MRI Scan session order

In both the pre- and post-intervention MRI sessions, scans were conducted in the following order: (1) resting-state during BOLD fMRI; (2) resting-state during pCASL; (3) emotion regulation task during fMRI; (4) T_1_-weighted structural scan; (5) magnetic resonance spectroscopy (MRS); and (6) T_1_-weighted TSE scan. The post-intervention session included three additional scans, which were performed between the ^1^H MRS and TSE scans in the following order: (1) ultimatum game task; (2) training-mimicking session during BOLD fMRI; and (3) training-mimicking session during pCASL. During both training-mimicking scans, participants engaged in their daily training without biofeedback (see below for details).

#### MRI Scan parameters

We employed a 3 T Siemens MAGNETOM Trio scanner with a 32-channel head coil at the USC Dana and David Dornsife Neuroimaging Center. T_1_-weighted 3D structural MRI brain scans were acquired pre and post intervention using a magnetization prepared rapid acquisition gradient echo (MPRAGE) sequence with TR = 2300 ms, TE = 2.26 ms, slice thickness = 1.0 mm, flip angle = 9°, field of view = 256 mm, and voxel size = 1.0 × 1.0 × 1.0 mm^3^, with 175 volumes collected (4:44 min). Functional MRI scans during resting-state, emotion-regulation, training and ultimatum-game tasks were acquired using multi-echo echo-planar imaging sequence with TR = 2400 ms, TE 18/35/53 ms, slice thickness = 3.0 mm, flip angle = 75°, field of view = 240 mm, voxel size = 3.0 × 3.0 × 3.0 mm. We acquired 175 volumes (7:00 min) for the resting-state scan and training scan, 250 volumes (10:00 min) for the emotion-regulation task and 244 volumes (9:45 min) for the ultimatum-game task. PCASL scans were acquired with TR = 3880 ms, TE = 36.48 ms, slice thickness = 3.0 mm, flip angle = 120°, field of view = 240 mm and voxel size = 2.5 × 2.5 × 3.0 mm^3^, with 12 volumes collected (3:14 min; 1st volume was an M0 image, 2nd volume was a dummy image that was discarded, and the remaining 10 volumes were five tag-control pairs) both during resting-state (pre and post intervention) and training-mimicking (post intervention) scans. The bolus duration is 1.517 s and the post-labeling delay is 1.8 s. This ASL approach provides high precision and signal-to-noise properties and has better test-retest reliability than pulsed or continuous ASL techniques^45^. The two-dimensional, multi-slice TSE scan was acquired with TR = 750 ms; TE = 12 ms; flip angle = 120°; bandwidth = 287 Hz/pixel; voxel size = 0.43 × 0.43 × 2.5 mm^3^, gap between slices = 1.0 mm, 11 axial slices). The ^1^H MRS data were acquired using a single-voxel point-resolved spectroscopy (PRESS) sequence with an echo time of 35 ms and repetition times of 2.0 s from a 4.1 cm^3^ (1.6 × 1.6 × 1.6 cm^3^) voxel localized to the anterior portion of the anterior cingulate cortex. Axial, sagittal, and coronal orientations were assessed for accurate voxel placement. Metabolite spectra were acquired with water suppression (water saturation pulse with bandwidth of 50 Hz) and 128 signal averages. Additionally, 6 water reference scans were acquired. The acquisition time of ^1^H MRS scan, including prescans, was approximately 5 min, and raw ^1^H MRS data were archived for processing offline. Each MRI session was approximately 60 min for pre-intervention and 90 min for post-intervention.

#### Pre- and post-intervention bold resting-state scan

Participants were instructed to rest, breathe normally and look at the central white cross on the screen.

#### Pre- and post-intervention pcasl resting-state scan

To assess whether the intervention affected blood flow during rest, in both MR sessions participants completed a second short resting-state scan. Participants were instructed to rest while breathing normally with their eyes open. To make visual inputs like those viewed during the training scan (for our analyses comparing rest vs. training scans), we presented red and blue circles alternately at a random rate (see Training sessions during BOLD and pCASL section below). Participants were asked not to pay attention to these stimuli.

#### Training-mimicking session during BOLD and pCASL

**Fig. 3:**
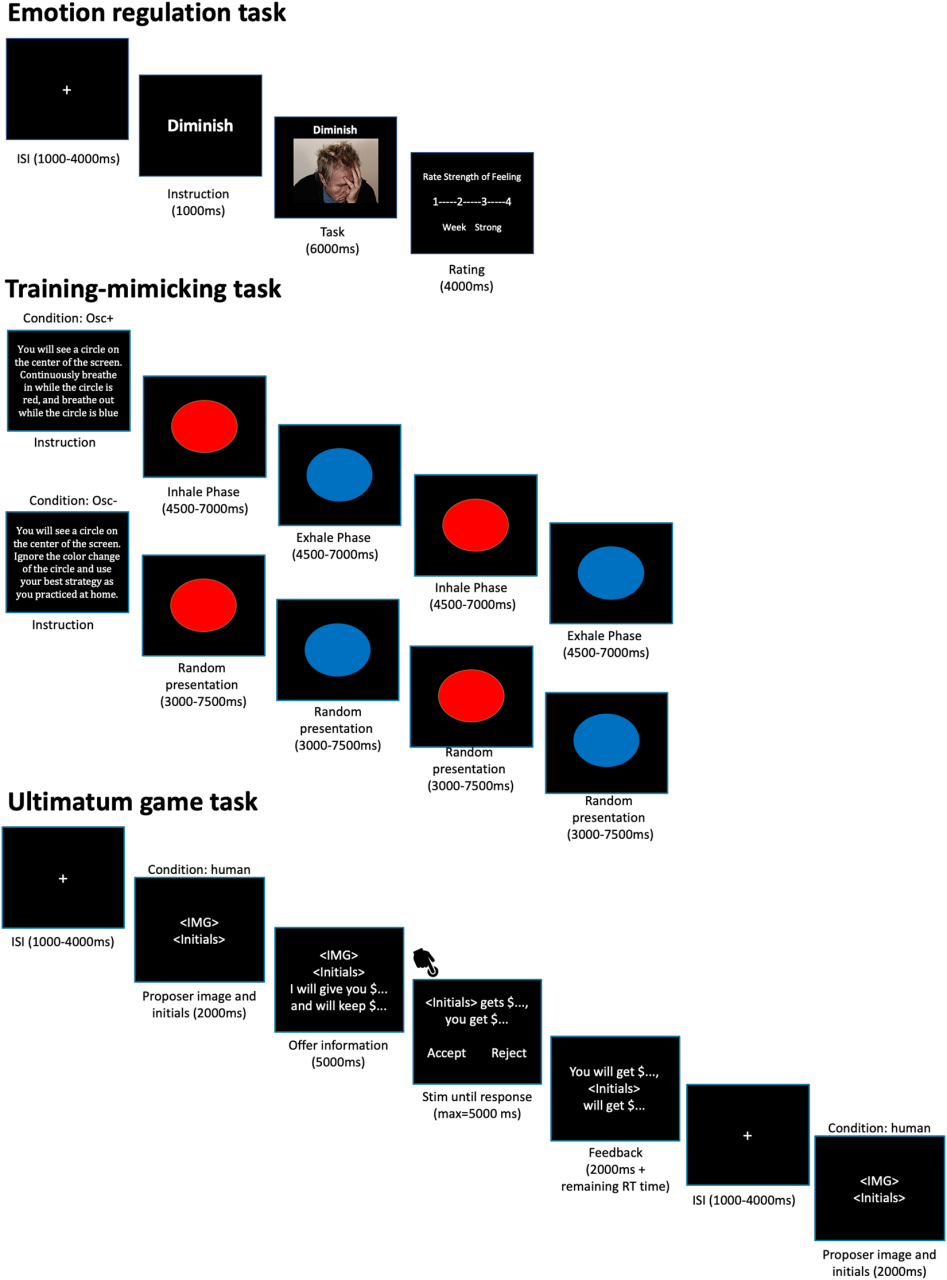
A visual representation of all experimental paradigms during task-based fMRI. ISI: inter-stimulus interval.

In the post-intervention scan session after the resting-state and emotion-regulation scans, participants completed their daily training without biofeedback during BOLD and pCASL scans. By this point, participants were well-trained, having each completed on average 57 training sessions at home. For the Osc+ group, a red and blue circle alternated at their resonance frequency. For example, if their resonance frequency was 12 sec, the red circle was presented for 6 sec followed by the blue circle for 6 sec. Participants were asked to breathe in with the red circle and breathe out with the blue circle. For the Osc- group, the stimuli were the same as for the Osc+ group; however, the red and blue circles alternated at a random rate and participants were told not to pay attention to them. See Fig. 3 for a visual representation of the training-mimicking session.

#### Emotion regulation task

Participants completed an emotion regulation task^46^ in the MRI scanner, which lasted for about 10 min. Each trial consisted of three parts: instruction (1 s), regulation (6 s), and rating (4 s). First, participants were given one of three instructions: “view”, “intensify,” or “diminish.” Then, during the regulation phase, they saw a positive, neutral, or negative image. Finally, they were asked to rate the strength of the feeling they were experiencing on a scale ranging from 1 (very weak) to 4 (very strong). See Fig. 3 for a visual representation of the emotion regulation task. For more details, see Min *et al*.^10^.

#### Ultimatum game task

During the week-7 MRI scanning session, participants completed an Ultimatum Game task^47^. Before scanning, participants were instructed that in this task they would be presented with offers proposed by other participants of the study (with each player making 4 offers) or offers randomly generated by the computer. Participants had the options to accept or reject the offers. If they accepted an offer, the money would be split as proposed by the other player. If they rejected an offer, both players would receive nothing on that trial. Participants were told that at the end of the study, one of the trials in the game would be randomly selected and both them and the proposer for that trial would be paid based on the participant’s response. In order to enhance realism, participants also played the role of proposer and were asked to make offers to future participants. The task lasted for about 10 minutes and consisted of 36 trials. Out of these, 18 included fair and 18 included unfair offers. Fair offers ranged from 0.40 to 0.55 of the endowment whereas unfair offers were defined as ones ranging from 0.05 to 0.20 of the endowment. Each trial lasted 14 seconds. At the beginning of each trial, the face and initials of the proposer for that trial were presented for a duration of 2 s. Then the offer proposed along with the face and initials of the proposer were shown for 5 s. The decision period was then followed, during which participants had 5 s to respond to the offer. Participants pressed one of two buttons on a button box to express their decision. Finally, the results screen was presented for a jittered duration of 2–6 s. In between trials, a fixation cross was shown on screen which lasted between 1–4 s. See Fig. 3 for a visual representation of the Ultimatum Game task.

Younger and older adults completed two slightly different versions of the task. In younger adults’ version of the task, half of the human proposers were proposers that players knew, while the other half were strangers. For familiar proposers, pictures of participants’ group mates were used. In older adults’ version of the task, all human proposers were strangers.

### Additional physiological measures during MRI

The physiological data collected during MRI scans include respiration, exhaled carbon dioxide (CO2), electrodermal activity, blood pressure, and heart rate. All the physiological data were collected at 10 kHz sampling rate using Biopac MP150 Data Acquisition System with MR-compatible sensors and recorded with AcqKnowledge software 5.0. Respiration was measured using the breathing belt, TSD201 transducer and transferred to the Biopac RSP100C module to be 0.05–1 Hz bandpass-filtered, amplified with 10 times of gain. Exhaled carbon dioxide (CO2) levels were measured using Philips NM3 Monitor (Model 7900) with nasal cannula and fed to Biopac MP150. The heart rate data were collected with a Nonin Medical 8600FO Pulse Oximeter and sent to Biopac MP150. The electrodermal activity was recorded using the Biopac GSR100C module. Blood pressure was measured using CareTaker device and software and recorded with Biopac MP150.

Tables 3, 4 summarize the main characteristics of physiological data collected during 7-minute resting-state BOLD MRI scans over two time-points. The original data files during MRI scans were stored using the Biopac AcqKnowledge software and later converted to a tsv file for sharing.

**Table 3:** Descriptive statistics of physiological measures during the resting scan.

Younger adults		Respiration rate (Hz) Week-2	Respiration rate (Hz) Week-7	Heart rate (bpm) Week-2	Heart rate (bpm) Week-7	ETCO_2_(mmHg) Week-2	ETCO_2_(mmHg) Week-7
Younger Osc+	N	54	46	59	45	35	41
	M (SD)	0.27 (0.06)	0.25 (0.07)	68.90 (9.65)	66.14 (8.69)	41.00 (3.13)	40.44 (3.27)
Younger Osc-	N	43	41	51	43	31	37
	M (SD)	0.29 (0.06)	0.29 (0.06)	69.79 (10.33)	67.97 (9.65)	40.56 (4.02)	40.76 (3.99)
**Older adults**		**Respiration rate (Hz) Week-2**	**Respiration rate (Hz) Week-7**	**Heart rate (bpm) Week-2**	**Heart rate (bpm) Week-7**	**ETCO**_**2**_**(mmHg) Week-2**	**ETCO**_**2**_**(mmHg) Week-7**
Younger Osc+	N	28	25	29	25	28	24
	M	0.23 (0.07)	0.22 (0.06)	65.87 (9.55)	62.11 (10.95)	39.97 (4.78)	40.11 (4.42)
Younger Osc-	N	24	24	27	21	25	25
	M	0.22 (0.06)	0.23 (0.06)	70.35 (11.78)	69.34 (13.59)	41.07 (4.04)	41.65 (4.30)

**Table 4:** Test-retest correlation of physiological measures during the resting scan.

Age group	Respiration rate (Hz)	Heart rate (bpm)	ETCO_2_(mmHg)
Younger adults	*r*(68) = 0.69, *p* < 0.001	*r*(80) = 0.53, *p* < 0.001	*r*(44) = 0.50, *p* < 0.001
Older adults	*r*(40) = 0.81, *p* < 0.001	*r*(41) = 0.89, *p* < 0.001	*r*(42) = 0.80, *p* < 0.001

### Blood collection and assay procedure

We previously reported details of the blood plasma collection and assay procedure^11^. During Week-1 and Week-6 lab visits, a phlebotomist drew 10 ml of blood from each participant’s arm into a K2 EDTA tube and then 2.5 ml of blood into a PAXgene RNA tube. The whole blood in the K2 EDTA tube was centrifuged at 1500 RPM for 15 minutes at room temperature (15 °C) to separate plasma from red blood cells. Plasma was stored in cryovials at −80 °C.

Plasma samples for both younger and older adults were analyzed using the automated Simoa SR-X analyzer with the commercially available Simoa Human Neurology 3-Plex A assay kit (Quanterix, Billerica, MA, USA) for Aβ42, Aβ40, and tTau. Plasma concentrations of pTau-181 were measured using the automated Simoa HD-X analyzer and the Simoa pTau-181 Advantage V2 kit (Quanterix, Billerica, MA, USA;see Min *et al*., 2023 for details)^11^.

## Data Records

The following data are available on the OpenNeuro data sharing platform (https://openneuro.org/datasets/ds003823)^48^. The files are organized in Brain Imaging Data Structure (BIDS) format^49^ (version1.5.0; http://bids.neuroimaging.io). BIDS is a data naming and organization system that facilitates the transfer, storage, and sharing of neuroimaging data. We used the BIDS validation tool provided by OpenNeuro to ensure that the dataset followed the BIDS system. Also, we anonymized T1-weighted scans by defacing them^50^.

At the root level of the dataset, participant demographic information, including sex, and handedness, and age group is provided in the participants.tsv file and these variables are further described in the accompanying data dictionary, participants.json. The participants.tsv file also indicates which of the different tasks, physiological data, and MRI scans are available for each participant at each time point. This information is organized into 33 columns containing “1” (data exist) or “0” (missing data) for all measures at each session (i.e., ses-pre_task-emotionRegulation; ses-post_task-emotionRegulation). Also, we organized the information about data quality in 15 columns containing “1” (recommend excluding) or “0” (recommend including) for MRI or physiological measures at each session. The json files for tasks, included at the root level of the dataset or in sub folders, provide the data dictionaries and any other metadata for tasks.

We organized the rest of the participants’ data in three ways: phenotype, subject folders, and derivatives folder^48^.

(1) “Phenotype”: This folder includes files that list all participants’ scores on standardized tests at each time point and participants’ responses to emotion questionnaires at each time-point (with one row per participant)

(2) “Sub- < ID > ”: This folder contains participants’ MRI scan data, physiological measures, and behavioral measures. Inside the folder of each participant with data available (i.e., n = 193 for total participants and n = 162 for longitudinal data, see also Fig. 1 for detailed information), there are two subfolders, named “ses-pre”, “ses-post” that contain data collected during pre- and post-intervention sessions, respectively. Another two subfolders, named “ses-calibration”, “ses-home,” contain heart rate measures collected during in-lab calibration sessions and during home practice sessions, respectively. The last subfolder, named “beh” has individual data for the picture memory tasks, which were administered in Weeks 4 and 5.

**Fig. 4:**
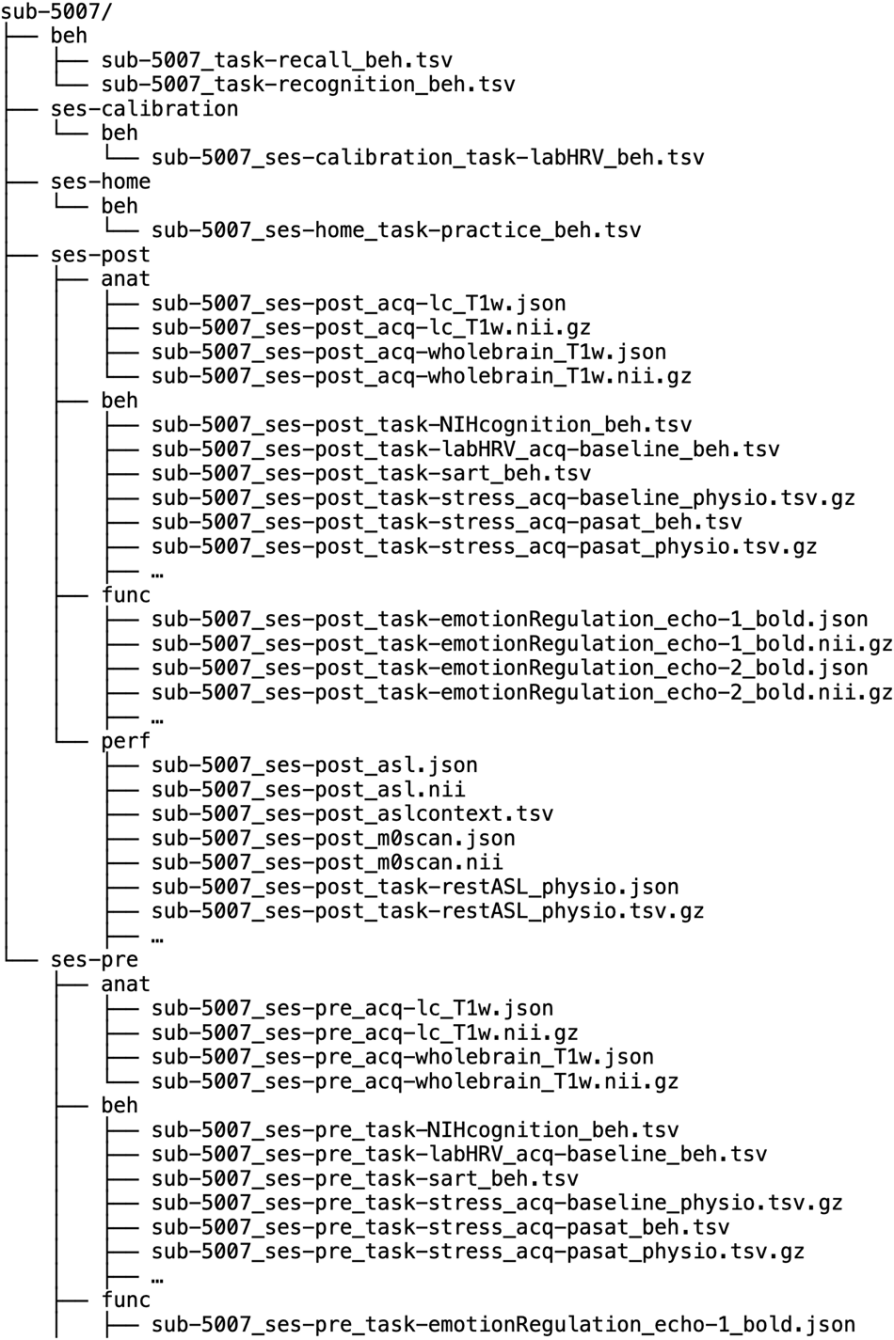
Example of the BIDS data structure for one participant. The data for subject 5007 are organized into five folders; two sessions for pre and post measurements, two sessions for HRV biofeedback data, one for calibration and the other for home training, and one last folder for behavioural data. While the data structure is consistent across subjects, there is some variation regarding data availability.

Inside “ses-pre” and “ses-post” folders, there are four subfolders named “anat”, “func”, “perf”, and “beh”. “Anat” folder contains T_1_-weighted structural images, “func” folder contains multi-echo BOLD scan data and the physiological data collected during those scans, “perf” folder contains pCASL scan data and the physiological data collected during those scans, and “beh” folder contains behavioral and physiological measures collected outside the scanner. Inside the “func” subfolder, there are files containing participant’s performance on the task (i.e., ‘events’ file), and physiological data for each task (i.e., ‘physio’ file) in addition to brain image files. The events file includes one row per trial, including onset time of each trial, duration of the event, trial type, response, response time, and the presented stimulus. Inside the “perf” subfolder, there are files containing (1) 10 tag & control acquisitions from the pCASL scan in a 4D file (“*_asl.nii”) and (2) an M0 calibration image from the pCASL scan in a 3D file (“*_m0scan.nii”)^51^. Figure 4 provides an example of the BIDS data structure for one subject. Table 5 provides detailed information about the file name and the location for each measure at each time point.

**Table 5:** Summary of data file name and location.

Category	Data type	Individual file name for time-point1	Individual file name for time-point2	Data summary file	Description
Demographics	Demographics			•participants.tsv	•Participants’ identification number, sex, age, handedness, and data present at each task at each time-point
Questionnaire	Self-reported behavior and emotion			•/phenotype/questionnaires_summary.tsv	•Summary of responses to emotion questionnaires (SAI, TAI, CESD, POMS, FFMQ, SRSI, CFQ, ERQ, PSS-10) and altruism scale
Sleep	Sleep time			•/phenotype/Whoop_Summary.tsv	•Sleep and HRV derived from slow-wave sleep, measured with WHOOP wristbands.
Cognitive task	NIH toolbox-cognition	•sub- < ID > /ses-pre/beh/sub- < ID > _ses-pre_task-NIHcognition_beh.tsv	•sub- < ID > /ses-pre/beh/sub- < ID > _ses-post_task-NIHcognition_beh.tsv	•/phenotype/NIH_Cognition_summary.tsv	•Performance on three tasks from NIH toolbox-Cognition; flanker test, list sorting working memory test, and pattern comparison processing speed test
Cognitive task	SART	•sub- < ID > /ses-pre/beh/sub-5006_ses-pre_task-sart_beh.tsv	•sub- < ID > /ses-posts/beh/sub-5006_ses-post_task-sart_beh.tsv	•/phenotype/SART_summary.tsv	•Performance on Sustained Attention to Response Task
Cognitive task	Memory task			•sub- < ID > /beh/sub- < ID > _task-recall_beh.tsv •sub- < ID > /beh/sub- < ID > _task-recognition_beh.tsv	•Recall and recognition on picture memory tasks at week4 and week5 lab visit
HRV- biofeedback	Calibration			•sub- < ID > /ses-calibration/beh/sub- < ID > _ses-calibration_task-labHRV_beh.tsv	•Heart rate data during lab calibration sessions; resting and several conditions included for each calibration session
HRV- biofeedback	Home training			•sub- < ID > /ses-home/beh/sub- < ID > _ses-home_task-practice_beh.tsv	•Heart rate data during home practice
Stress task	Physiological data	•sub- < ID > /ses-pre/beh/sub- < ID > _ses-pre_task-stress_acq-baseline_physio.tsv.gz •Same for acq-pasat, stroop, and recovery	•sub- < ID > /ses- post/beh/sub- < ID > _ses-post_task-stress_acq-baseline_physio.tsv.gz •Same for acq-pasat, stroop, and recovery		•Physiological data (ECG, respiration, continuous blood pressure, and skin conductance response) to assess reactivity to and recovery from acute stress
Stress task	Behavioral data	•sub- < ID > /ses-pre/beh/sub- < ID > _ses-pre_task-stress_acq-pasat_beh.tsv •sub- < ID > /ses-pre/beh/sub- < ID > _ses-pre_task-stress_acq-stroop_beh.tsv	•sub- < ID > /ses-post/beh/sub- < ID > _ses-post_task-stress_acq-pasat_beh.tsv •sub- < ID > /ses-post/beh/sub- < ID > _ses-post_task-stress_acq-stroop_beh.tsv		• Behavioral responses during PASAT and strop task to induce acute stress
MR Imaging	Functional-resting	•sub- < ID > /ses-pre/func/sub- < ID > _ses-pre_task-rest_echo-1_bold.nii.gz •Same for echo-2 and echo-3	•sub- < ID > /ses-post/func/sub- < ID > _ses- post_task-rest_echo-1_bold.nii.gz •Same for echo-2 and echo-3		•Multi-echo fMRI •BOLD resting-state scan
MR Imaging	Functional-resting-state ASL	•sub- < ID > /ses-pre/perf/sub- < ID > _ses-pre_asl.nii	•sub- < ID > /ses-post/perf/sub- < ID > _ses-post_asl.nii		• pCASL resting-state scan
MR Imaging	Functional-Emotion regulation	•sub- < ID > /ses-pre/func/sub- < ID > _ses-pre_task-emotionRegulation_echo-1_bold.nii.gz •Same for echo-2 and echo-3	•sub- < ID > /ses-post/func/sub- < ID > _ses-post_task-emotionRegulation_echo-1_bold.nii.gz •Same for echo-2 and echo-3		•Multi-echo fMRI data •Emotion regulation task during BOLD scan
MR Imaging	Anatomical-T_1_	•sub- < ID > /ses-pre/anat/sub- < ID > _ses-pre_acq-wholebrain_T1w.nii.gz	•sub- < ID > /ses-post/anat/sub- < ID > _ses-post_acq-wholebrain_T1w.nii.gz		•T_1_-weighted structural scan
MR Imaging	Biochemistry-MRS	•/derivatives/MRS/sub- < ID > /ses-pre/*.IMA	•/derivatives/MRS/sub- < ID > /ses-post/*.IMA	•/derivatives/MRS/MRS_summary.tsv	•proton magnetic resonance spectroscopy
MR Imaging	Anatomical-TSE	•sub- < ID > /ses-pre/anat/sub- < ID > _ses-pre_acq-lc_T1w.nii.gz	•sub- < ID > /ses-post/anat/sub- < ID > _ses-post_acq-lc_T1w.nii.gz		•two-dimensional, multi-slice TSE scan
MR Imaging	Functional-UG		•sub- < ID > /ses- post/func/sub- < ID > _ses-post_task-ultimatumGame_echo-1_bold.nii.gz •Same for echo-2 and echo-3		•Multi-echo fMRI data •Ultimatum Game task during BOLD scan
MR Imaging	Functional-training mimicking		•sub- < ID > /ses-post/func/sub- < ID > _ses-post_task-trainingMimicking_echo-1_bold.nii.gz •Same for echo-2 and echo-3		•Multi-echo fMRI data •Training mimicking session during BOLD scan
MR Imaging	Functional-training-mimicking ASL		•sub- < ID > /ses-post/perf/sub- < ID > _ses-post_task-trainingMimickingASL_m0scan.nii		•Training mimicking session during pCASL scan

(3) “derivatives”: This folder contains MRS, mriqc, and freesurferQC folders. Inside the MRS folder, there is a “MRS_summary.tsv” file and individual IMA files, which are readable by dicom readers, under individual subject folders. “MRS_summary.tsv” includes the individual metabolite concentration levels and quality metrics for all scans for all participants. Also, information including the voxel orientation information extracted from the rda header in the IMA file are included in the file. Inside each subject folder, there are two subfolders, named “ses-pre”, “ses-post” including raw MRS data as IMA file format. The pre session included one MRS scan, which produced three ‘.IMA’ files. During the post session, some participants had two MRS scans; the first MRS scan occurred at the same point in the scan sequence as the pre MRS scan. The second, optional, MRS scan was completed after all other task scans were done. Inside the mriqc folder, there are multiple files for scan types; “group_T1w_mriqc.tsv” and “group_BOLD_mriqc_ < task-name > .tsv” include the quality control metrics for the T_1_-weighted and functional (BOLD) MRI scans, respectively. Inside the freesurferQC folder, there is a freesurfer_QC.tsv file including Freesurfer quality metrics with outlier participants on these metrics flagged.

## Technical Validation

In this section, we describe quality control metrics. We take a conservative approach for data exclusions. We generally did not exclude brain imaging data unless (1) the MRI scan session was interrupted by unexpected events (e.g., an earthquake or power outage), (2) an absence of a usable T_1_-weighted scan due to technical error or scan terminated by participants, or (3) incidental findings. Also, we did not exclude behavioral or physiological data unless (1) the task was interrupted by unexpected events (e.g., an earthquake or power outage) or (2) obvious sensor error or data input error due to a technical issue. But we applied quality control for data analyses and shared the results of quality control metrics in the derivative folder and quality control results in the participants.tsv file. This way, the future users of the datasets can use the quality-controlled data we recommend including, evaluate our quality control methods, or apply their own quality control methods on the datasets. Importantly, this places the responsibility for inclusion and exclusion of data in the hands of the users of the datasets.

### MRI Data quality assessment

The quality control metrics for the T_1_-weighted and functional (BOLD) MRI scans were computed by the MRIQC package, which outputs several quality control metrics as well as a report with visualizations of different aspects of the data. The quality control metrics for T_1_-weighted images are stored in the group_T1w.tsv in the derivatives/mriqc folder^48^. The quality control metrics for the functional scans are stored in derivatives/mriqc folder.

#### T_1_-weighted scans

**Fig. 5:**
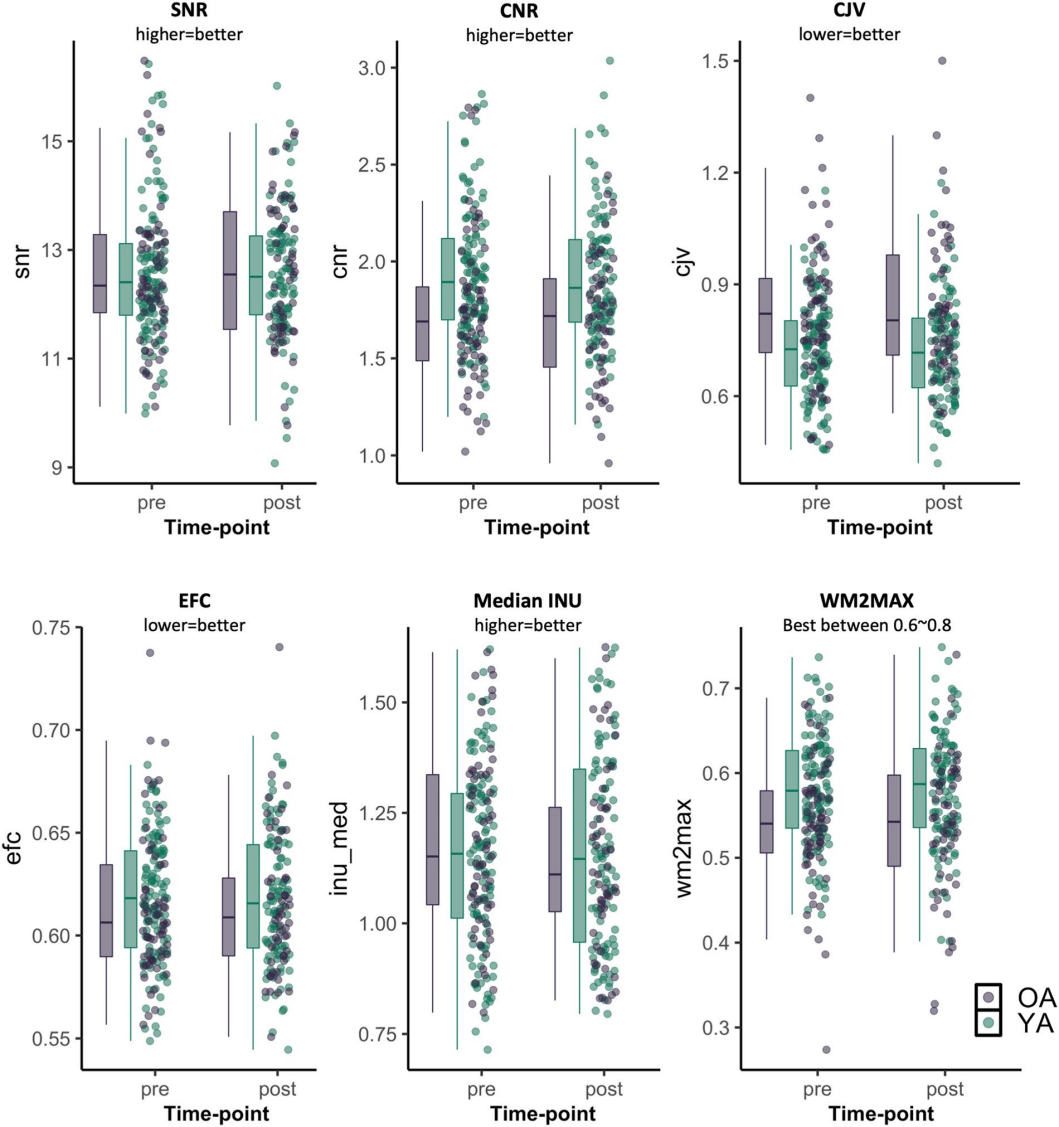
Quality control metrics related to the T_1_-weighted scans at each time-point. SNR: signal-to-noise ratio; CNR: contrast-to-noise ratio; CJV: coefficient of joint variation, an index reflecting head motion and spatial inhomogeneity; EFC: entropy-focused criterion, an index reflecting head motion and ghosting; Median INU: intensity non-uniformity, an index of spatial inhomogeneity; WM2MAX: ratio of median white-matter intensity to the 95% percentile of all signal intensities.

Using the MRIQC pipeline, we obtained quality control metrics as well as a report with visualizations of different aspects of the data for all T_1-_weighted scans. We visually checked all individual subject reports for artifacts including reconstruction errors, failure of defacing, and segmentation. We considered defacing successful if the 3D render did not contain more than one partial facial feature (eyes, nose, or mouth) and no brain tissue had been removed during defacing^52^. In Fig. 5, we visualize several quality control metrics related to the T_1_-weighted scans over two time-points (pre and post). MRIQC includes the signal-to-nose ratio (SNR) calculation proposed by Dietrich *et al*.^53^, using the air background as a noise reference. Additionally, for images that have undergone some noise reduction processing, or the more complex noise realizations of current parallel acquisitions, a simplified calculation using the within tissue variance is also provided. Higher values indicate better quality. The contrast-to-noise ratio (CNR)^54^ is an extension of the SNR calculation to evaluate how separated the tissue distributions of GM and WM are. Higher values indicate better quality. The coefficient of joint variation (CJV) of GM and WM was proposed as an objective function^55^ for the optimization of intensity non-uniformity (INU) correction algorithms. Higher values are related to the presence of heavy head motion and large INU artifacts. The entropy-focus criterion (EFC)^56^ uses the Shannon entropy of voxel intensities as an indication of ghosting and blurring induced by head motion. Lower values are better. Median INU is an index of spatial inhomogeneity. It estimates the location and spread of the bias field extracted^57^. The smaller spreads located around 1.0 are better. The white matter to maximum intensity ratio (WM2MAX) is the median intensity within the WM mask over the 95% percentile of the full intensity distribution, that captures the existence of long tails due to hyper-intensity of the carotid vessels and fat. Values should be around the interval [0.6, 0.8]^58^. In general, data quality appears consistent across time. All quality control metrics related to the T_1_-weighted scans for each participant, including those visualized in Fig. 5, are stored in the group_T1w_mriqc.tsv file in the derivatives/mriqc folder^48^. Here we do not exclude any subjects based on IQMs, but subsequent researchers can use the available IQMs to exclude scans as they see fit.

Each participant’s T_1_-weighted structural images were preprocessed using Freesurfer image analysis suite version 6.0 (http://surfer.nmr.mgh.harvard.edu/). Cortical reconstruction and volumetric segmentation were performed. Following initial preprocessing, we used the Freesurfer 6.0 image analysis suite longitudinal stream to automatically extract volume estimates^59^. After completing the longitudinal Freesurfer pipeline, we used automated measures computed by FreeSurfer of the contrast-to-noise ratio (the difference in signal intensity between regions of different tissue types and noise signal) and the Euler number (a metric of cortical surface reconstruction) to identify poor quality structural scans (Chalavi *et al*. 2012; Rosen *et al*. 2018). For analyses of volumetric change, we identified outliers (N = 4 for younger adults and N = 2 for older adults) who on a box-and-whisker plot were above Q3 + 3 × the interquartile range on either of these metrics on either pre or post images. Freesurfer quality metrics and the list of outliers are provided in the freesurfer_QC.tsv file in the derivatives/freesurferQC folder^48^.

#### Functional (BOLD) scans

**Fig. 6:**
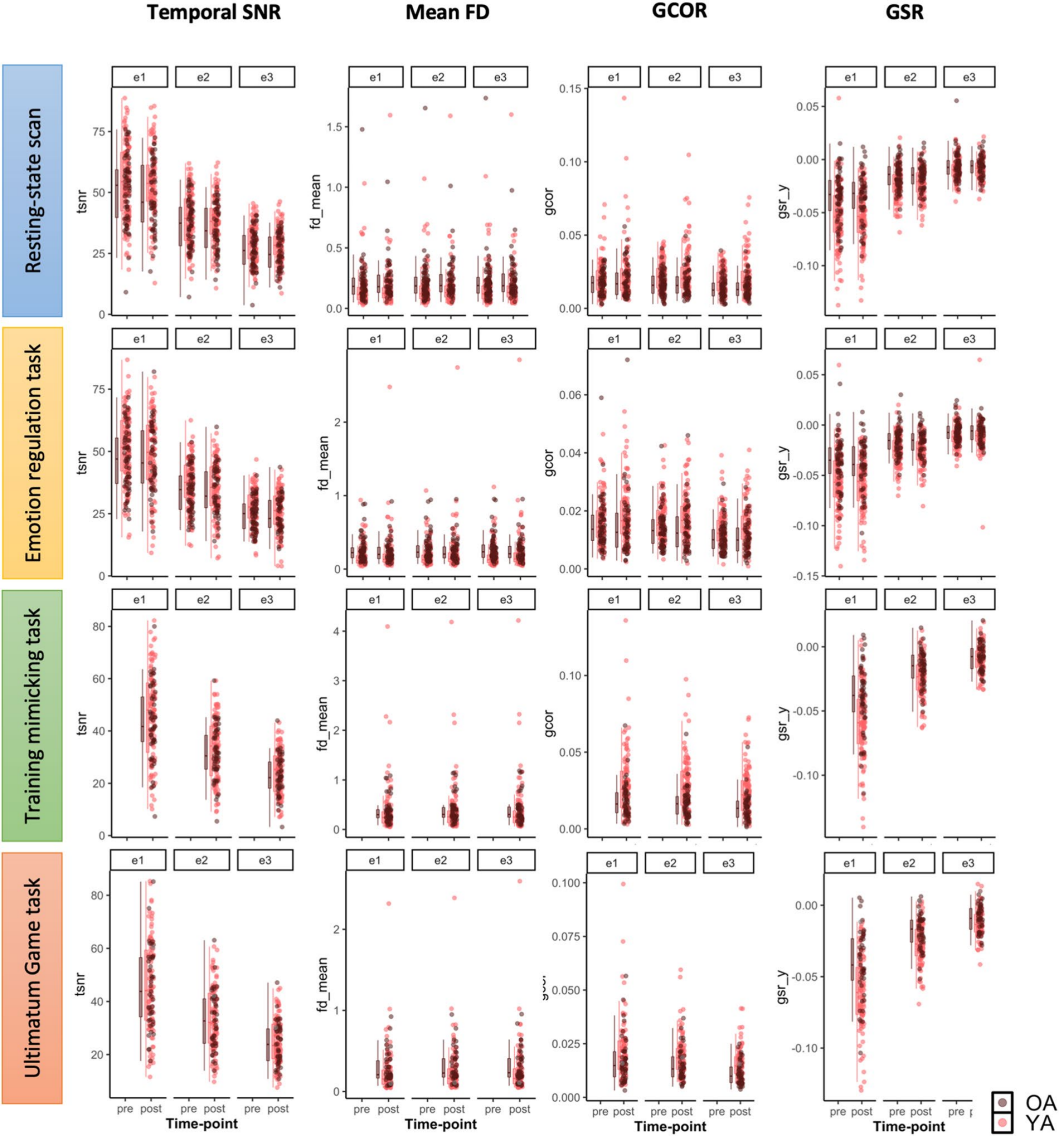
Quality control metrics related to the multi-echo functional (BOLD) scans at each time-point for resting-state scan, emotion regulation task scan, training mimicking task scan, and UG task. SNR: signal-to-noise ratio, an index of signal quality; FD: framewise displacement, an index of overall movement; GCOR: global correlation, an index of the presence of global signals; GSR: ghost-to-signal ratio, an index of ghosting along the phase-encoding axis.

We ran the functional (BOLD) scans through the MRIQC pipeline and visually checked the resulting reports were visually checked for artifacts including reconstruction errors, registration issues, and incorrect brain masks. In Fig. 6, we visualize several quality control metrics related to the functional scans across three echo times (e1 = 18 ms, e2 = 35, and e3 = 53 ms) over two time-points (pre and post). Temporal SNR (tSNR) is a simplified interpretation of the tSNR definition^60^. The MRIQC pipeline provided the median value of the tSNR map calculated as, tSNR = ⟨S⟩_t_/σ_t_, where ⟨S⟩_t_ is the average BOLD signal (across time), and σ_t_ is the corresponding temporal standard-deviation map. Higher values are better when comparing scans at the same echo (differences across echo times are expected due to effects of echo time on BOLD contrast). Mean Framewise Displacement (FD) is a measure of subject head motion, which compares the motion between the current and previous volumes. Higher values indicate lower quality. Global Correlation (GCOR) is the average correlation of all pairs of voxel time series inside of the brain. GCOR measures differences between data due to motion/physiological noise/imaging artifacts as well as global neural fluctuations^61,62^. MRIQC measures ghost-to-signal ratio (GSR) along the x or y encoding axes. Higher values indicate lower quality. Like the T_1_-weighted quality control metrics, the functional quality metrics appear consistent across time. All quality control metrics related to the functional (BOLD) scans for each participant, including those visualized in Fig. 6, are provided in the group_BOLD_mriqc.tsv file in the derivatives/mriqc folder^48^. Here we do not exclude any subjects based on IQMs, but subsequent researchers can use the available IQMs to exclude scans as they see fit.

#### MRS scans

**Fig. 7:**
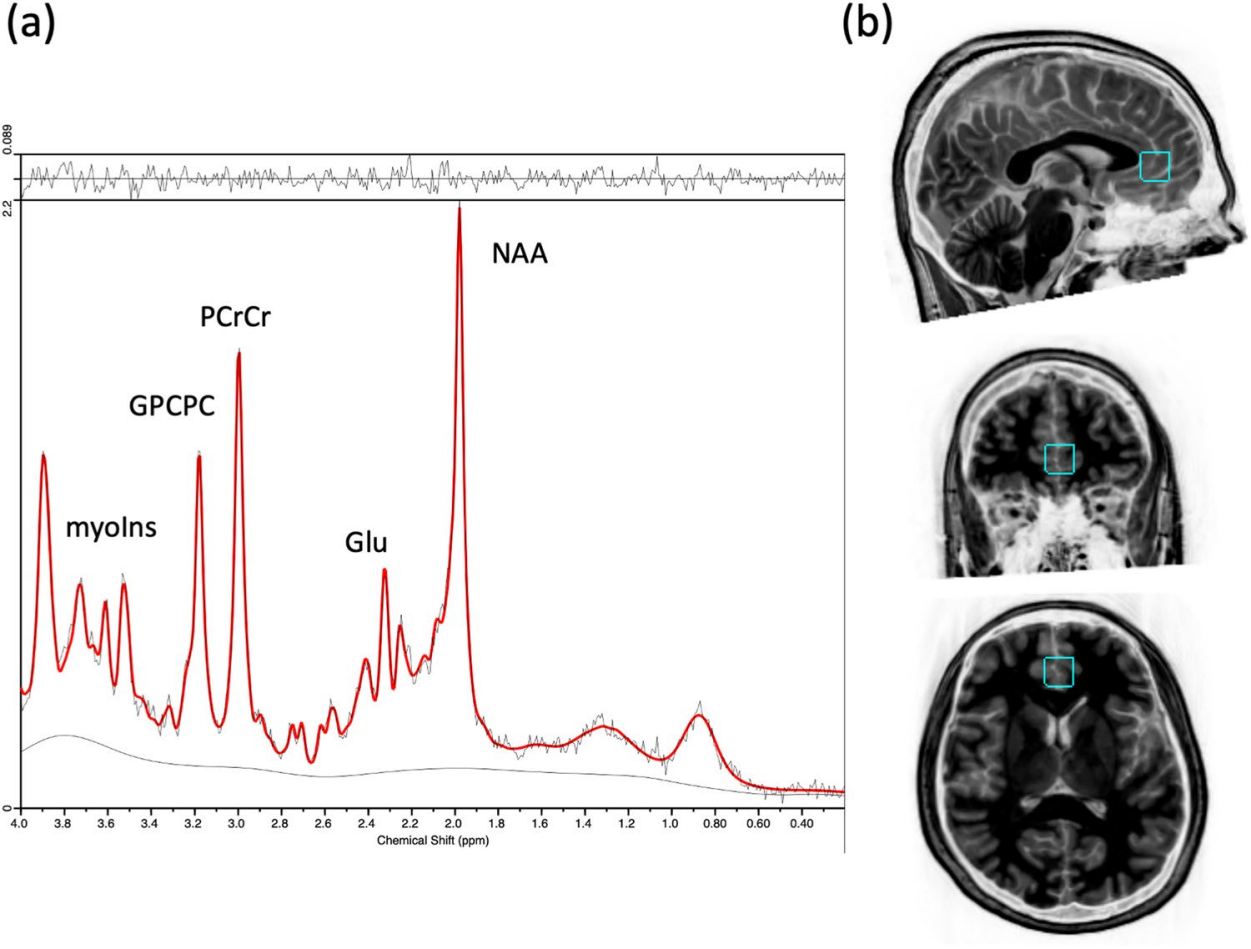
An example of an individual quantified MRS spectrum (**a**) and the sagittal, coronal and axial view of the MRS voxel placed in the anterior cingulate cortex, from top to bottom (**b**).

Post-processing and quantification of the ^1^HMRS data were 100% automated^63^. For each ^1^H MRS spectra, the metabolites N-acetyl-aspartate (NAA), phosphocreatine plus creatine (PCr + Cr), trimethylamines [glycerophosphocholine plus phosphocholine (GP + CPC)], and myo-inositol, glutamate, and glutamine (as well as the less reliable metabolites, aspartate, gamma-aminobutyric acid, glutathione, lactate, n-acetylaspartylglutamate, scyllo-inositol and taurine) were quantified using the Linear Combination (LC) Model software^64^ with a simulated basis set for the a priori knowledge reflecting the acquisition parameters. An example of an individual MRS spectrum from the ^1^H MRS voxel placed in the anterior cingulate cortex is shown in Fig. 7. Freesurfer and FSL tools (FLIRT, FAST, MRI_VOLSYNTH, MRI_VOL2VOL) were used to tissue segment the T_1_-weighted images, which were then used to quantify the tissue fraction values within each voxel location.

**Fig. 8:**
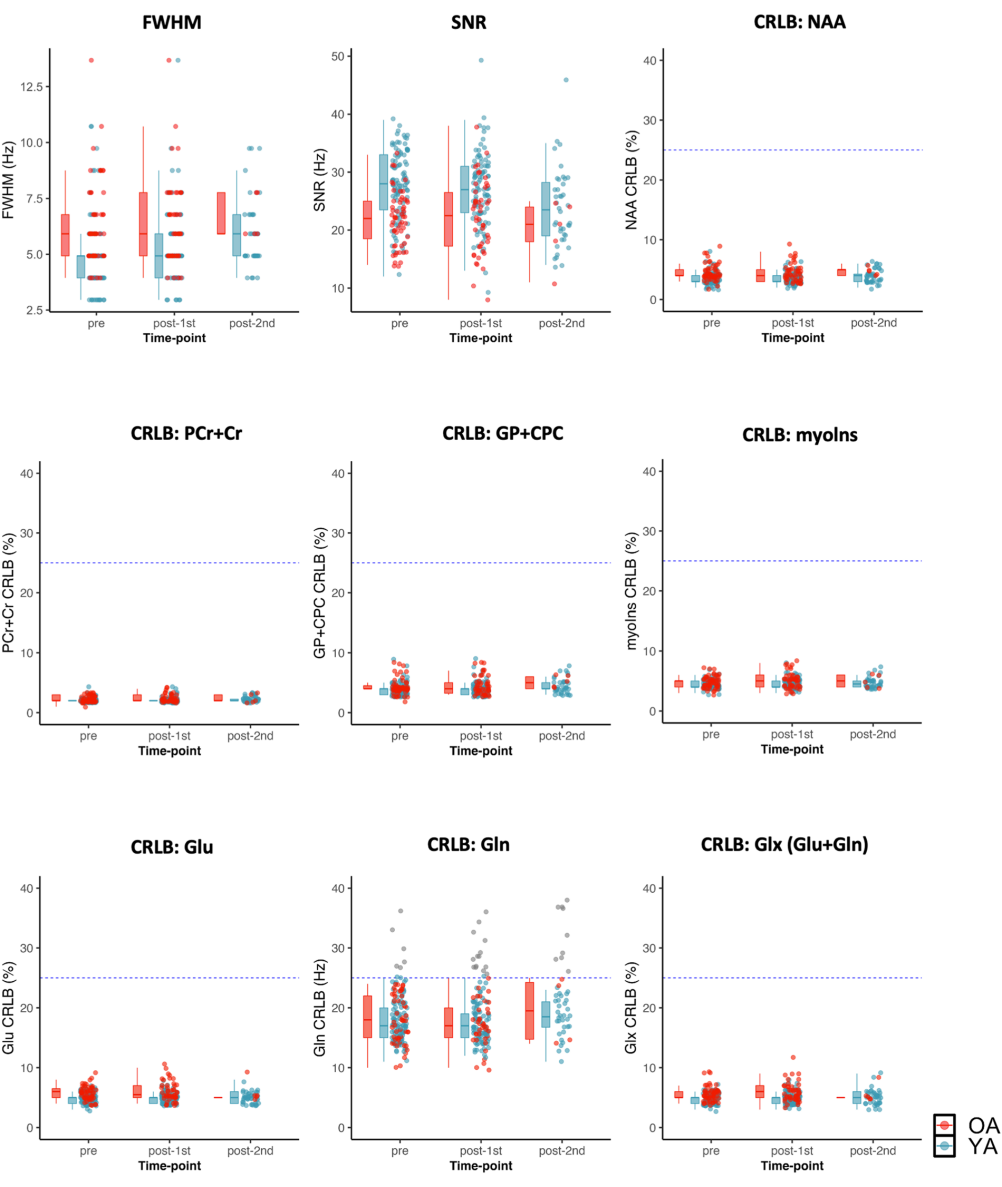
Quality control metrics related to the MRS scans at each time-point. 9 out of 353 spectra were rejected as bad quality and not included in the figure. The 25% Cramér-Rao lower bound (CRLB) is indicated with dotted line across the figure for each metabolite. Those outlier values for which CRLB > 25% are indicated with gray color. FWHM: full width at half maximum of singlet peaks; SNR: signal to noise ratio; CRLB: Cramér-Rao lower bound; NAA:N-acetyl-aspartate; PCr + Cr: phosphocreatine plus creatine; GP + CPC: glycerophosphocholine plus phosphocholine; myoIns: myo-inositol; Glu: glutamate; Gln: glutamine; Glu_Gln: Glu + Gln.

As quality control metrics for the MRS scans, we used SNR, line width reflecting the full width at half maximum (FWHM) of NAA, and Cramér-Rao lower bound (CRLB)^65^. Nine MRS spectra were rejected for poor quality (pre: 3, post-1st: 3, post-2nd: 3) due to extreme CRLB values. The distribution of quality metrics are visualized in Fig. 8 after this removal of poor quality data. Also, all metabolite levels that have CRLB higher than 25% or another chosen threshold are tagged “outlier” with gray color in Fig. 8. We included all data with quality tags in the shared data file, “MRS_summary.tsv”^48^, to allow future users of the datasets to apply their own threshold on the datasets^66^. The individual metabolite levels and quality metrics are provided for all scans for all participants in the MRS_summary.tsv file in the derivatives/MRS folder^48^.

### Resting heart rate data quality assessment

**Fig. 9:**
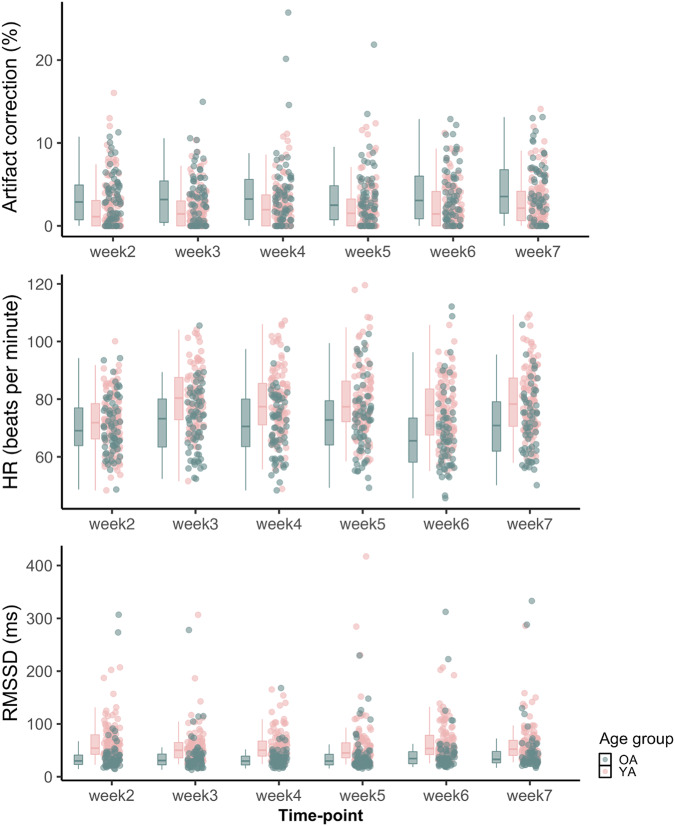
Distribution of artifact correction (%), heart rate (HR), and RMSSD during rest from weekly lab calibration sessions.

The resting heart rate data was measured during weekly HRV calibration sessions. The pulse was measured with an infrared pulse plethysmograph (ppg) ear sensor and the interbeat interval (IBI) data was extracted after eliminating ectopic beats or other sources of artifacts through a built-in process in emWave pro software. Figure 9 depicts distributions of the artifact correction rate, mean heart rate, and RMSSD.

### Quality assessments for other measures

To check test-retest reliability, we reported the intraclass correlation coefficients and typical percentage error^67^ of emotion questionnaires in Table 2 and test-retest correlation of physiological measures in Table 4. Blood plasma analyses were performed in duplicates and mean % coefficient of variation [%CV]of Aβ42, Aβ40, tTau, and pTau were reported previously^11^.

## Data Availability

Code for collecting, formatting, and processing the data is available at https://github.com/EmotionCognitionLab/HRV-ER-dataset_release and https://github.com/EmotionCognitionLab/emWave_HRV. Information about the code dependencies and package requirements are available in the same Github repository.
